# Harveian Oration 2025: Nephrology 1950–2000: An exciting journey from birth to maturity

**DOI:** 10.1016/j.clinme.2025.100549

**Published:** 2025-12-25

**Authors:** John Feehally

**Affiliations:** University of Leicester, University Road, Leicester, Leicestershire, LE1 7RH, United Kingdom

## Introduction

Nephrology was late to emerge as a clinical specialty. It did not exist in 1950, but then followed major technical and clinical developments, resulting by 2000 in a specialty whose structure and organisation are familiar to contemporary patients and clinical staff.

1950–2000 was the era of greatest change in nephrology and greatest risk, when much was demanded of patients and their families. An era of pioneering giants on whose shoulders we stand. An era which nephrology might not have survived, because there were unexpected vicissitudes, and some peers thought our pioneers ill-judged.

## Richard Bright

Although this is a story of the second half of the 20th century, it was a great 19th-century figure, Richard Bright of Guy’s Hospital, who drew the novel conclusion, based on clinical findings and post-mortem examination of kidney tissue. that people with ‘the dropsy’ (ie oedema), along with protein in their urine, had kidney disease. Bright also noted that those same subjects had ‘hardening of the pulse with cardiac enlargement’; Bright had identified hypertensive cardiovascular disease, 40 years before the first clinical measurement of blood pressure. In 1950, almost nothing had changed since Bright in our understanding of kidney disease, nor were there any meaningful treatments. There were no thiazide or loop diuretics, no tolerable anti-hypertensives, no dialysis, no transplantation. Irreversible kidney failure had a 100% mortality.

## Manchester in the 1950s

But there was a nucleus for the study of kidney disease in the UK in 1950, in Manchester, where the professor of medicine, Robert Platt (later PRCP)^1^ gathered round him some world-class physician scientists interested in cation and anion handling by the kidney. They included Douglas Black PRCP,^2^ Malcolm Milne FRS,^3^ and Oliver Wrong.^4^ For treatment, they could offer adjustments of ion and fluid intake, but nothing more. Not really clinical nephrologists, Black dubbed them ‘intellectual nephrophiles’.

In 1950 the Renal Association was founded^5^ – a very small group of those interested in the kidney, of whom only a handful were physicians. Then two developments began a revolution in kidney investigation and treatment.

## Renal biopsy

Using renal biopsy, it was at last possible to look at kidney disease while it was happening, rather than waiting for autopsy.^6^ From the 1960s, Stewart Cameron, reprising the work of his Guy’s predecessor Richard Bright, began to report, using Kaplan–Meier survival analysis, the natural history of each of the morphological patterns of glomerular disease revealed by renal biopsy. The early use of renal biopsy produced a flurry of intellectual interest for nephrologists and pathologists, but little new treatment.

## Steroids for nephrotic syndrome

One treatable glomerular disease had been identified before the arrival of renal biopsy. In 1952 from Glasgow came the first report that many children with nephrotic syndrome respond to cortisone.^7^ We still use corticosteroids in nephrotic children to this day, though regrettably we still do not understand their mechanism of action.

This unfortunately led to a rather indiscriminate use of steroids in glomerular disease, resulting in limited responses and extensive toxicity, but also provided the setting for the first randomised controlled trial in all nephrology – a trial in the UK of corticosteroids in adults with nephrotic syndrome.^8^

## Dialysis

Intermittent haemodialysis was first used in the UK in 1946 for temporary support in reversible acute renal failure (ARF). However, non-dialysis management of ARF was in vogue at that time – careful fluid and electrolyte care combined with a high-calorie, zero-protein (and very nauseating) diet (to minimise uraemia). It was more than a decade before dialysis began to establish its current role in the management of ARF.

But the far bigger question was whether dialysis could be a chronic, indeed lifelong, therapy for people with irreversible kidney failure, end-stage renal disease (ESRD). Belding Hibbard Scribner in 1960 in Seattle was the first to treat ESRD with haemodialysis,^9^ having solved the problem of repeated high flow rate vascular access using the arteriovenous shunt, which was soon replaced by the arteriovenous fistula.^10^

The first chronic dialysis programme beyond Seattle was established by Stanley Shaldon^11^ at the Royal Free Hospital. Very soon other UK centres offered chronic dialysis, notably led by Hugh de Wardener^12^ at Charing Cross Hospital, David Kerr at Newcastle-upon-Tyne,^13^ and Stewart Cameron^14^ at Guy’s Hospital, London. It was soon obvious that there were more patients than could be treated in the limited space being set aside for dialysis in hospitals. In 1964, Shaldon established the first home dialysis patient in Europe, who used a converted spare bedroom, although others often needed a portacabin in the back garden to act as a dialysis room. Home dialysis was much used in the UK to avoid overwhelming hospital dialysis facilities. There was no precedent for such sustained commitment by patients and their carers – two (later three) dialysis treatments a week at home, each lasting up to 10 hours. Treatment would be lifelong unless a transplant was possible.

This very demanding treatment was made more tolerable by the patient support structure available: nephrology had invented the multiprofessional clinical team, years before it was part of care in other specialties. Nurses, technicians, dietitians, pharmacists and more were all empowered. Nurses in particular took the lead in delivering regular haemodialysis.

## Dialysis under challenge

Dialysis worked; it was here to stay. New units were opening with no budget and no designated funds. There was public and media pressure to expand dialysis. But there was professional scepticism. An anonymous *Lancet* editorial saw dialysis only as an expensive means of ‘averting death’ rather than offering rehabilitation. An opinion challenged by a young medical student, who was on dialysis, Robin Eady^15^ who undertook home haemodialysis for 24 years before receiving a renal transplant which was functioning at his death 30 years later. He became a highly respected academic dermatologist, a tribute to the rehabilitation offered by renal replacement therapy.

A Department of Health committee chaired by Hugh de Wardener in 1964 recommended 20 dialysis units across the UK, and these were funded. But any optimism was soon dispelled.

## Complications of dialysis

### Hepatitis

In the 1960s and early 1970s there were outbreaks of viral hepatitis in several dialysis units; patients and staff were infected and there were deaths.^16^

These hepatitis outbreaks were an existential threat to the growth of haemodialysis in the UK, an open goal for the sceptics. The cause was hepatitis B, but the proximate cause was severe overcrowding and equipment sharing with the good intention of dialysing as many treatable people as was possible.

Dialysis units moved to quell and prevent these epidemics. Infected patients were segregated, were transplanted if at all possible, or moved to home dialysis. A key figure in protecting dialysis from closure was Max Rosenheim PRCP,^17^ who chaired an enquiry into hepatitis in renal units. His 1972 report was transformative, defining measures needed to prevent future outbreaks, the ‘universal precautions’ which remain to this day the basis of routine infection prevention.

### Aluminium

In the 1970s ‘dialysis dementia’ appeared, geographically clustered cases of rapid cognitive decline with fracturing osteodystrophy, which was caused by aluminium poisoning.^18^ Dialysis exposes patients to high volumes of tap water, and some water authorities were adding large amounts of aluminium sulphate to clear particulates. This problem is prevented by reverse osmosis, deionising the water, now a standard feature of dialysis facilities.

### Dialysis amyloid

In the 1980s, troublesome musculoskeletal symptoms in long-term dialysis patients were soon attributed to amyloid deposits associated with beta-2 microglobulin,^19^ which was too large (MW 11,700) to be freely dialysed. Happily this dialysis-associated amyloid has reduced in prevalence by using dialysis membranes with higher molecular weight cut-offs.

### Metabolic bone disease

Also emerging were the consequences not of dialysis itself, but of the unnatural state of being kept alive without kidney function. Dialysis did not, of course, restore the endocrine functions of the kidney. The final hydroxylation step of vitamin D was lost, and the deficiency of active vitamin D along with the phosphate retention in renal failure drove complex metabolic bone disease – with hyperparathyroidism and osteomalacia, often with vascular calcification.

### Erythropoeitin

Healthy kidneys also produce erythropoietin (Epo). Epo-deficient and Epo-resistant dialysis patients were typically enduring haemoglobin levels around 6–8 g/dL. Blood transfusion worked only transiently, due to the shortened red cell half-life in uraemia. Multiple transfusions created risk of iron overload, and also of transfusing anti-HLA antibodies, which might compromise transplant options. But little else could be offered until the mid-1980s, when recombinant human erythropoietin became available and produced a prompt and sustained improvement in haemoglobin levels.^20^ Patients were transformed in energy and wellbeing. Of course there were matters to resolve – not least the cost. But Epo worked.

### Cardiovascular consequences of dialysis

A complication of long survival with ESRD was cardiovascular disease, first associated with kidney disease by Richard Bright. By the turn of this century, it was clear that the life expectancy of a 25-year-old on dialysis was the same as that of a 75-year-old in the general population,^21^ this difference mostly attributable to cardiovascular disease. More than an acceleration of atheroma, there was also an intractable cardiovascular disease characterised by vascular and valvular calcification, and myocardial fibrosis, a continuing major problem of ESRD.

## Academic nephrology

Although dialysis, its consequences and complications began to dominate the clinical work, nephrology had been an academic specialty from the time of Platt, and it remained so. Renal Association meetings were dominated by tubular physiology and experimental immunology, but gradually broadened to represent the whole gamut of clinical science, reflecting the broader research interests of the kidney community.

The early giants also recognised the need for a kidney-specific research fund, launching the National Kidney Research Fund in 1966, which in 2005 became Kidney Research UK.^22^ The intellectual energy of the UK nephrology community remains strong, and across a broad swathe including physiology, immunology, genetics, epidemiology and clinical trials – UK nephrology has made world-class contributions.

## Children

Children with kidney failure were not within the brief of de Wardener’s 1964 committee. Paediatric nephrologists in the 1970s developed a national plan based on regional centres, which the Department of Health did not fund, and few children were being treated. Frustrated paediatric nephrologists began to appear on national media, pointing out that treatable children were dying for lack of facilities, but there was still no funding. Then one day the prime minister (Margaret Thatcher) was being interviewed about a *cause célèbre* of the day, a man who needed a bone marrow transplant but was unable to get it because of where he lived. Cornered, she announced on TV that the government was planning national networks of specialist centres to treat uncustomary diseases. Although there was no evidence of any such planning, within days funds became available for regional paediatric nephrology centres.

## Transplantation

Kidney transplantation is the best treatment for ESRD, for those who are fit enough. In many centres in the 1960s and 1970s, dialysis and transplantation had developed separately. The surgical techniques for kidney transplantation were well established; the challenge was achieving sufficient immunosuppression to prevent and control rejection without severe adverse effects.

It was soon realised that renal units would be overwhelmed by the number of dialysis patients unless an active transplant programme became conjoined. The size of the prevalent dialysis population would only stabilise when the number leaving dialysis (because of death or transplantation) matched the number starting dialysis (including those returning to dialysis after transplant failure). Dialysis and transplant services gradually became integrated, though there was some jousting as powerful clinical leaders learnt to be partners.

Early transplant outcomes however were very poor, apart from the fortunate few with renal failure who had an identical twin who did not share an inherited kidney disease, and could be a donor.

In the 1960s, the only immunosuppression to prevent rejection was high-dose corticosteroids; 1 year graft survival (GS) was <5%, which increased to 30% with the introduction of azathioprine. The change to lower-dose steroid protocols obviated many of their side effects, but 1 year GS only reached 50–60%.

A major step forward was the introduction in the late 1970s of the calcineurin inhibitor cyclosporin,^23^ enabling 1 year GS around 85%, with incremental improvement since then. The introduction of cyclosporin however was not without challenge, as the therapeutic window between effective immunosuppression and nephrotoxicity proved to be very narrow, due to intrarenal vasoconstriction.

## Supply and demand

In the 1980s we knew that we were undertreating renal failure, although the term ‘rationing’ was not in the NHS lexicon. The daily reality was that spaces on the dialysis units were always filled, with more patients waiting, and additional dialysis units were opening too slowly. The addition of peritoneal dialysis as a home-based alternative to haemodialysis only provided modest mitigation.

Then in 1990 the Renal Association, with Netar Mallick as president, decided that it could no longer only be a scientific and educational society; it must also advocate for renal services to government. In 1991 Mallick, with other RA leaders, met the secretary of state for health, and achieved a written commitment that there would be treatment guaranteed for 40 new patients per year per million population age <65 years. Inadequate, but since that first guarantee had been given, funding for the sequential growth of dialysis has been negotiated.

Crucial to this long-awaited success was availability for the first time of peer-reviewed published multicentre incidence data to support the case of need.

## Digital information systems

Clinical nephrology has always been a ‘numbers’ discipline. Serum creatinine and other serial laboratory parameters are powerful in measuring clinical progress and highlighting problems. In the 1960s, Hugh de Wardener was first to realise the power of numbers for all aspects of dialysis care, and develop digital systems to make this possible. By the mid-1980s, the great majority of UK renal units were using a digital system, many years before other specialties, and it was soon impossible to imagine how we had managed clinical care without it.

But more power could come from the combining of data from multiple units in a national registry – driving up standards of care through comparative audit, research and resource planning. From a pilot in the early 1990s, within a decade there was a nationwide UK Renal Registry,^24^ covering all patients on renal replacement therapy in all centres, and then expanding to all patients under nephrology care. The continuing success of the Registry has been facilitated by financial sustainability through capitation funding, and by keeping it in the ownership of the Renal Association.

## Ethical challenges

The work of nephrologists has been entwined in shifting ethical dilemmas.^25^ At the beginning, who were the few who should be offered this complex, demanding and – as it turned out – life-prolonging treatment? Seattle had an unsatisfactory experiment in which a lay committee decided which of the many desperate patients were given dialysis. We do not know exactly how the ‘Lucky 13’ at the Royal Free were chosen, but Shaldon later indicated his view of those best suited to the rigours of long-term dialysis: including stable marriage, their own home, private transport and sufficient income; all notions that sit uncomfortably with our modern egalitarian instincts.

As dialysis expanded, there were difficult decisions about who should not receive treatment even if it was technically possible, especially when dealing with unrealistic patient and family expectations.

Nephrologists have all had discussions over the years with patients who have bravely fought but have had enough, who realise that their dialysis is miserably prolonging the end of their life, who are ready to stop. And by mutual consent – patient, family, nephrologist, clinical team – we no longer dialyse, we manage terminal uraemic symptoms and death follows within a week or so. However we describe those actions, which I believe to be ethical, we need a term for it other than ‘assisted dying’, so sullied by the current debate.

## Conclusion

Nephrology is mature. From nothing in 1950, it is now a nationally funded, high-cost, highly audited service, with a research-active culture. Nephrology was the first specialty to replace the function of a completely failed solid organ, and to sustain complex home-based therapy. It was the inventor of the multiprofessional clinical team, and the first specialty to release the power of digital information.

But the hepatitis outbreaks and the scepticism of an old guard had nearly derailed everything. By comparison, a 21st century of steady consolidation is welcome.

It is important to consider whether, in our modern, risk-averse NHS, dialysis and transplantation would have become established, given their early poor results and severe complications. I suspect the answer is that they would not.

The ‘giants’ whom I have named were characterised by high intellect, high drive, and high passion for the work; by a lack of self-importance, and a generosity of spirit. They were interested in me; if I cared much about nephrology, and wanted to commit all of my limited gifts to it, they went above all that might reasonably be expected of a mentor to support me. We should reflect on the sacrifices that those giants and their families made – no talk of ‘work–life balance’ then. Nephrology did not emerge by serendipity; it was driven into life and nurtured to maturity by giants. I used to meet the giants at Renal Association meetings. After exacting scientific discussion we mingled with them in the pub, there was a buzz of conversation and encouragement and collaboration. In a small specialty, giants who were our heroes soon became our mentors and then our friends a camaraderie which we should still cherish and learn how to sustain in our different modern medical world.

## Further information

An extensive archive of the history of nephrology in the UK can be found at UK Kidney History www.ukkidneyhistory.org.

## Funding

This research did not receive any specific grant from funding agencies in the public, commercial or not-for-profit sectors.

## CRediT authorship contribution statement

**John Feehally:** Writing – review & editing, Writing – original draft, Visualization, Validation, Supervision, Software, Resources, Project administration, Methodology, Investigation, Funding acquisition, Formal analysis, Data curation, Conceptualization.

## Declaration of competing interest

The authors declare that they have no known competing financial interests or personal relationships that could have appeared to influence the work reported in this paper.
